# ‘If we don’t assess the patient’s vision, we risk starting at the wrong end’: a qualitative evaluation of a stroke service knowledge translation project

**DOI:** 10.1186/s12913-022-07732-w

**Published:** 2022-03-16

**Authors:** Torgeir S. Mathisen, Grethe Eilertsen, Heidi Ormstad, Helle K. Falkenberg

**Affiliations:** 1 National Centre for Optics, Vision and Eye Care, Faculty of Health and Social Sciences, University of South-Eastern Norway, Hasbergs vei 36, 3616 Kongsberg, Norway; 2 USN Research Group of Older Peoples’ Health, University of South-Eastern Norway, Drammen, Norway; 3 Department of Nursing and Health Science, Faculty of Health and Social Sciences, University of South-Eastern Norway, Drammen, Norway

**Keywords:** Stroke, Vision assessment, Visual impairments, Knowledge translation, Implementation, Rehabilitation, Outcomes

## Abstract

**Background:**

Visual impairments (VIs) affect 60% of stroke survivors and have negative consequences for rehabilitation and quality of life poststroke. Symptoms of VIs post stroke are difficult to identify for stroke survivors and health care professionals without using a structured vision assessment. In this study, we qualitatively evaluate the implementation outcomes after implementing a structured visual assessment with the Competence, Rehabilitation of Sight after Stroke Vision (KROSS) assessment tool in stroke care services.

**Methods:**

This is a qualitative study comprising four focus group interviews. The health care personnel (HCP) involved in the implementation or with experience using the KROSS assessment tool in practice were invited to participate. We used Proctor et al.’s definitions of implementation outcomes as a framework, which informed the interview guide and analysis. We used a deductive - inductive content analysis, as described by Elo and Kyngäs.

**Results:**

The participants found the structured vision assessment with the KROSS tool as being acceptable; they expressed a motivation and intention to use the new routine in practice. They believed it was important to assess their patient’s visual function because it influenced other rehabilitation activities and activities of daily living. Most of the participants reported having adopted the vision assessment in their practice, except for those participants from the home care services who experienced that they have few stroke survivors to follow up on. The assessment was believed to be more appropriate to perform within the rehabilitation services where there is more of a focus on functional assessments. Although vision assessment was new to all the participants, they felt that they improved their vision assessment skills by regularly using the assessment tool. Together with sufficient instructions and supervision, they believed that vison assessment was feasible for their practise. Including the vison assessment in the existing routines and systems was important to promote sustainable implementation.

**Conclusion:**

Implementing a structured vision assessment with the KROSS tool in health care services was experienced as acceptable and feasible. The new routine led to increased attention towards poststroke VIs and increased collaboration with vision experts. Tailoring the routine to each practice and how they organise their work can support the integration of a vision assessment in their routines. To promote better vision care poststroke vision assessment and follow up should be included in the stroke care pathways.

**Supplementary Information:**

The online version contains supplementary material available at 10.1186/s12913-022-07732-w.

## Background

Vision impairments (VIs) are common poststroke and affect approximately 60% of all stroke survivors [1]. VIs poststroke include visual field defects, eye movement disorders, reduced visual acuity and different visual perceptual disorders [2, 3]. Poststroke VIs have negative consequences for quality of life, mobilisation and rehabilitation outcomes and are associated with depression and reduced activity [4–8]. Despite this, there is a lack of attention given to assessing visual functions in stroke care and within clinical guidelines [9–12]. Stroke survivors experience that their VIs are overlooked by health care professionals in contrast to other consequences after stroke, such as limb palsy or aphasia, and are offered limited support and follow-up [8, 10, 13]. Frequent vision problems after stroke are blurred, altered and reduced vision, visual field loss, diplopia and a variety of perceptual problems [14, 15]. These problems may cause difficulties with reading, trouble finding things, walking into objects and more [10, 15, 16]. Although some will immediately become aware of their impaired vision, almost 40% of stroke survivors with stroke-related VIs do not report visual symptoms in the acute stroke unit [14]. Hence, a present visual impairment may remain undetected and unnecessarily negatively influence rehabilitation and quality of life after stroke [9, 16, 17]. To identify VIs after stroke, visual functions need to be properly assessed [16, 18]. Currently, no tools that include the assessment of vision and common visual functions affected by stroke are systematically used in Norwegian stroke care. In the UK, the Vision Impairments Screening Assessment (VISA) tool was developed to screen stroke survivors for VIs. With the VISA tool, health care personnel (HCP) in the stroke unit without formal competence in vision and eye care can identify VIs and appropriately refer patients to further vision assessments [19]. In Norway, a similar tool, the Competence, Rehabilitation of Sight after Stroke (KROSS) assessment tool, has been developed and tested in two stroke units and used by multidisciplinary HCP to assess vision poststroke and promote a follow-up for VIs [20, 21]. The KROSS vision assessment tool consists of objective assessments of visual acuity, eye movements, visual field, visual attention and reading, questions for identifying subjective symptoms, and observations in activities of daily living (ADL). The symptom questions are both general, asking for experiences of changes in the patient’s vision, and more specific related to the visual functions. The tool has 17 items, scored as yes/no there is an identified problem, and 4 items related to information to the patient. All persons identified with a problem are referred for further assessment.

The current study is an evaluation of the KROSS Knowledge Translation project (KROSS KT), a project to implement a structured vision assessment and follow-up of VIs poststroke among municipal health care services [10, 20, 22]. In collaboration with a Norwegian municipality and patient organisations, we adapted the KROSS tool and competence workshop to a municipal context [20] and implemented it in three municipal health services frequently used by stroke survivors [23]: the inpatient rehabilitation unit, home rehabilitation and home care. As the KROSS KT project progressed, other health care services wanted to be a part of the implementation, attend the workshop and use the KROSS tool. Hence, a specialist rehabilitation hospital and stroke unit located in the municipality were included. We used the knowledge to action (KTA) model as the framework for the implementation [24]. The implementation strategies used in the KROSS KT project were chosen as a result of assessments of barriers and facilitators to implementing a structured vision assessment in municipal health care services, which have been described in an earlier study [20]. We used multicomponent initiatives that combined dissemination, education, collaboration with researchers and knowledge users, incentives and facilitation [25]. More details about the KROSS KT project are described in an earlier publication about the barriers and facilitators to the implementation of a structured vision assessment in the municipality [20].

There are many ways to evaluate implementation. In the present study, we have used the implementation outcomes described by Proctor et al. [26] in qualitative focus group interviews. To facilitate a common language for evaluating implementation, Proctor et al. review the literature on evaluation and describe and define the implementation outcomes [26]; they define implementation outcomes as ‘the effects of deliberate and purposive actions to implement new treatments, practises and services’ [26]. The implementation outcomes were used as guidance and structure in the current study’s interviews and analyses when evaluating the implementation of a structured vision assessment using the KROSS tool. The outcomes are *acceptability, adoption, appropriateness, feasibility, penetration, sustainability, fidelity* and *costs* [26].

The current study’s aim is to evaluate the implementation as experienced by the HCPs involved in the KROSS KT project, here as anchored in Proctor et al.’ implementation outcomes.

## Methods

### Design

In the present qualitative study, we used focus group interviews for the data collection. We used the Consolidated Criteria for Reporting Qualitative Research (COREQ) checklist to promote transparent reporting [27].

### Participant selection

HCP with experience of being in the KROSS KT project were contacted and invited to participate in the focus group interviews. Recently, we described the expected barriers and facilitators to the implementation of KROSS in municipal health care services, identifying the contextual differences between the municipal services. The participants experiences of having a flexible work schedule or not, degree of time constraints and competence especially affected their views on the likelihood of successful implementation [20]. When monitoring knowledge use during implementation, all services reported differences in their use of the KROSS tool and new vision routines in their practise. Therefore, we chose to create focus groups based on the participants’ affiliations to the services they worked in. We also considered that this way of organising the groups would contribute to a more free expression of experiences that would be independent of the ‘successfulness’ of the implementation. The participants who responded to our invitation and consented to participate in the study were allocated into four focus groups. Group 1 included home care services nurses (*n* = 2). Group 2 included municipal rehabilitation unit nurses and physiotherapists (*n* = 5). Group 3 included specialist rehabilitation hospital occupational therapists, sports pedagogues, physiotherapists and neuropsychologists (*n* = 9). Group 4 was a mix including one nurse from home-based rehabilitation, two case handlers (nurses) and one physiotherapist from the local hospitals stroke unit (*n* = 4).

### Setting

The focus group interviews took place on the services’ premises (special rehabilitation hospital and municipal rehabilitation unit) or at the university (home care and mixed group), here based on the participant’s preferences. The first author, who is the project manager, acted as the moderator during the interviews. Most of the participants and the moderator were acquainted with each other because of having worked before this on the earlier parts of the KROSS KT project. It was made clear that the purpose of the study was not to evaluate the participants themselves but instead to discuss their experiences with the implementation and structured vision assessment. Each group was interviewed once 16–18 months after the implementation started.

### Data collection

The interviews lasted from 40 to 70 min and were digitally recorded and transcribed verbatim by the first author. We developed an interview guide to cover Proctor et al.’s implementation outcomes [26]. In addition, topics that arose during the implementation phase were addressed, such as the participants’ experiences of performing the tests or interpretations of the KROSS manual. The participants were also encouraged to speak freely about their experiences of participating in the project and using the KROSS tool in their services (the interview guide is available as supplementary file 1).

### Analysis

We analysed the data using a content analysis with a deductive - inductive approach, as described by Elo and Kyngäs [28]. They described an analysis process containing a preparation phase, an organising phase and a phase reporting the process and results. The transcripts were analysed by two researchers (TSM and HKF, a nurse and an optometrist, respectively). We used NVivo 12 to manage the data [29]. In the preparation phase, the material was thoroughly read by both researchers to become familiar with the data. In the organising phase, we used a matrix based on Proctor et al.’s eight implementation outcomes [26], where TSM and HKF individually reviewed and categorised the data according to the implementation outcomes. During the analysis, the researchers met frequently to discuss the data and which implementation outcome the data fit into. Differences were discussed until consensus was reached. Once all meaning units were assigned to an implementation outcome, the principles of inductive content analysis were used to develop categories within the bounds of each implementation outcome. This Elo and Kyngäs [28] described as unconstrained analysis. (See Table 1 for an overview of the analysis).

**Table 1:** An example of the analysis process from deductive to inductive content analyses

	Step 1 Deductive content analysis	Step 2 Inductive content analysis	Step 3 Inductive content analysis
Proctor et al.’s eight implementation outcomes [26]	Data reviewed for content and coded for correspondence with or exemplification of the implementation outcomes	Create sub-categories	Conceptualizing and abstracting into categories
I. Acceptability Definition: The perception among implementation stakeholders that a given treatment, service, practice or innovation is agreeable, palatable or satisfactory	*It is easier to perform visual assessments now, as we learned something concrete to use for the assessment. This makes it easier to have an opinion about visual function.* (G3) *It was very useful to listen to and engage with the stroke survivors, who shared and explained how their vision loss affected their everyday life. I think this was great.* (G2)	Access to the KROSS tool was considered important to perform vison assessment Real stories from stroke survivors promotes motivation	A motivating and useful KROSS workshop

The data within each implementation outcome were analysed and grouped into sub-categories and categories. The final categories were discussed and agreed upon by all the authors. The outcomes and included categories are presented in Table 2. The implementation outcome—costs—was not a specific focus in the current study, although aspects about the use of resources are discussed in some of the other implementation outcomes.

**Table 2:** The categories from the analysis are presented in the right column and implementation outcomes with its definitions in the left [26]

Implementation outcome and definition	Categories
**Acceptability** *The perception among implementation stakeholders that a given treatment, service, practice or innovation is agreeable, palatable or satisfactory.*	• A motivating and useful KROSS workshop • Acceptance of prioritising a vision assessment in the hectic workday • Vision assessments create a positive change for the patients
**Adoption** *The intention, initial decision or action to try or employ an innovation or evidence-based practice.*	• Differences in the extent of knowledge use • Increased awareness of visual impairments in clinical practise
**Appropriateness** *The perceived fit, relevance or compatibility of the innovation or evidence-based practice for a given practice setting, provider or consumer and/or perceived fit of the innovation to address a particular issue or problem.*	• Assessing vision is a first step to better vision care • More appropriate in a rehabilitation setting
**Feasibility** *The extent to which a new treatment or an innovation can be successfully used or carried out within a given agency or setting.*	• Practise makes perfect • Helpful instructions and supervision • Integration of the KROSS tool into the medical records ease documentation • Limited time available
**Fidelity** *The degree to which an intervention was implemented as prescribed in the original protocol or as intended by the programme developers.*	• Followed the KROSS protocol but did not test all patients
**Penetration** *The integration of a practice within a service setting and its subsystems.*	• Vision assessment now included in service allocation office case handling • Visual function assessment integrated into the clinical awareness • More structured interdisciplinary collaboration with vision experts
**Sustainability** *The extent to which a newly implemented treatment is maintained or institutionalised within a service setting’s ongoing, stable operations.*	• Integration into existing routines • Desire for formal vision competence

## Results

A total of 17 categories were identified during the analysis, and for each of the implementation outcomes, there was a variation between two and four categories (see Table 1).

### Acceptability

There were three categories in the data related to the participants’ perceptions of acceptability in the KROSS KT project.

#### A motivating and useful KROSS workshop

The participants expressed that overall, they were happy to be part of the implementation project. The workshop provided new knowledge about VIs after stroke that they could start to use in their clinical practice immediately. Learning about the extent of VIs following a stroke and its significant impact on life poststroke gave the participants motivation and understanding that it was useful to implement vision assessments into their practise.

*I think that vision after stroke is very neglected, so it is good to start with this now. I reckon that it is true that to see is important; to avoid falling over and hurt yourself or break something. And we can’t see it, if they have poor vision, if they can see or not. And the patients do not say anything about it either. (G1)*

The combination of theory and practical training together with the personal experiences expressed by the stroke survivors gave the workshop credibility and acceptance for the implementation.

*It was very useful to listen to and engage with the stroke survivors, who shared and explained how their vision loss affected their everyday life. I think this was great.* (G2)

Practicing the assessment tool on stroke survivors under supervision during the workshop made them confident that they would be able to use it in their practise.

*It is easier to perform visual assessments now, as we learned something concrete to use for the assessment. This makes it easier to have an opinion about visual function. (G3)*

#### Acceptance of prioritising a vision assessment in the hectic workday

All the participants expressed that they had already experienced a high workload in their current practise. Adding the KROSS tool as a new routine had been a trade-off with other work, which some experienced as a dilemma when having to choose between equally important tasks. An important factor for choosing to use the KROSS tool was that they experienced how knowledge of the patient’s visual function was beneficial in, for example, ADL and mobilisation.

*Well, we need to think about how we can defend the extra use of time. I feel that sometimes, the patient either gets physical training, or they get their vision assessed. And what is most important? In some cases, perhaps both are equally important. To facilitate physical training better, however, the vision must have been assessed.* (G2)

The participants reported that using the KROSS tool regularly reduced the assessment time. Some said that the assessment took around 20 min if it had been a long time since they performed their last vision assessment. The participants had different opinions on what they considered an acceptable use of time; most considered 15 to 20 min as being acceptable.

*Usually, we schedule one hour per home visit because we have many things to assess. This (KROSS) took 20 minutes last time I did it; I don’t think that’s too much. (G4)*

*I think I am using about 20 minutes, which might be because I am going through the papers during the assessment and need too… it’s the same with other assessments too. The more you do them, the easier they get. And you notice things easier and such. (G2)*

#### Vision assessments create a positive change for the patients

The participants experienced that all the patients they had tested so far appreciated the added vision assessment. In some cases, if there was a complex outcome and the patient was exposed to comprehensive assessments after the stroke, the vision assessment was postponed to reduce the strain on the patient.

*Patients are very interested [to be tested with KROSS]. They are often very positive about the additional assessment. Most people are concerned about their own health.* (G4)

*Before this project, I knew some patients who had been in despair because they had vision problems they could not make head or tail of. And where nobody would follow this up. So I do think it is important to identify vision problems. And this [KROSS tool] is great to use. Yes, it is.* (G3)

### Adoption

Two categories represent adoption. The services reported differences in their extent of using the KROSS routine. Some had integrated it into their regular routines, some when they expected a visual problem, and a few did not use it at all. Despite their differences in using the KROSS tool, all groups expressed that their overall attention to VIs had improved both for themselves and among colleagues and overall in the health care services involved in the KROSS KT project.

#### Differences in the extent of knowledge use

All the participants stated that they intended to start assessing vision among stroke survivors using the KROSS tool after the workshop. However, not all the participants had managed to implement the vision assessment, and the adoption differed between the services. In the municipal rehabilitation unit and home rehabilitation, they now assessed nearly all stroke patients in their service, whereas HCP working in the home care services said they had not been able to use the KROSS assessment tool because they had not yet been seeing any stroke survivors in their services.

*Ehh... but sadly, we’ve not been able to use it afterwards [the workshop]. Because… but we have some more focus on it and think about it occasionally. However, we have not seen any stroke patients yet. (G1)*

In the specialist rehabilitation hospital, they assessed all their stroke patients with the KROSS tool, and in the local hospital, they used the KROSS tool if they suspected that the patient might have a vision problem because either the patient reported a visual problem or the HCP made clinical observations that indicated a visual problem, like walking into things or neglecting one side.

*Yes, I have assessed most stroke patients. Of course, it has happened that I have forgotten some and suddenly think about it when the patient no longer receives our services. But I try to assess all stroke patients. (G3)*

Of the participants who regularly performed the test, most had attended the workshop. The other HCP had been trained to use the KROSS tool by their colleagues who had participated in the KROSS workshop. The participants were encouraged to carry out peer training to allow more patients to be assessed. Peer training was especially common in the specialist rehabilitation hospital, but also in home-based rehabilitation.

*A colleague, in addition to me, now performs the KROSS assessments. It took some time to feel confident to do the assessment, but we did it together the first time. So now there are more than just me. I think that is smart. (G3)*

#### Increased awareness of visual impairments

All the participants emphasised that taking part in this implementation project had increased the attention of VIs poststroke in their services, including home care services.

*Even though I have not had any stroke patients yet, I have been thinking about it a lot since the workshop. That we need to be aware of possible vision problems. (G1)*

It was not only the workshop attendees themselves who reported an increased awareness to VIs, but they also said their colleagues were now asking for a vision assessment of their patients.

*Participant 1: Often, my colleagues on the team remind me. ‘Should we do the KROSS test on this patient?’*

*Participant 2: It is like that for us too, the others remember because they are more with the patients. (G4)*

The KROSS project provided them with a tool and knowledge to help identify vision problems and separate them from other problems. Some symptoms of VIs they described earlier could be misinterpreted as a symptom of cognitive difficulties or related to communication problems they now considered if such symptoms could be related to changes in visual function. Knowledge of the patients’ visual function made them more confident in some clinical judgements compared with before implementation.

*And when it comes to cognitive function, if we don’t assess the patient’s vision, we risk starting at the wrong end. Vision should be assessed on day one, actually. (G3)*

### Appropriateness

Two categories represent the participants’ expressions of appropriateness. Their experiences of appropriateness were connected to how they believed that assessing vision could contribute to an improvement of vision care after stroke and their amount of engagement with stroke survivors in their daily work.

#### Assessing vision is a first step to better vision care

During implementation, there was a clear referral pathway for patients identified with VIs. This was something the participants highlighted as important. Although they wished they could include vision rehabilitation in their services or quickly refer their patients to such rehabilitation, they all recognised the importance of the initial assessment to identify a potential problem. Many had missed such standard pathways for patients with VIs before implementation.

*I’m now thinking of how to follow up vision after a stroke. One thing is proper correction with glasses and other, more basic things. However, there are some problems beyond that. If there are problems with eye-movement control or perceptions. We have experienced that there are no follow-up to refer to….* (G3)

The participants felt that it was satisfying to be able to identify vision problems using the KROSS tool. However, some experienced that it was a problem that their services did not offer vision rehabilitation while working with the patients because none of the services included any eye care specialists. Those patients identified with VIs were referred to an external ophthalmologist or optometrist. However, they wanted to be able to start vision rehabilitation while the patient was in their care to promote visual function and rehabilitation.

*We want to be able to do something with what we find. We want to start training that can be continued in later stages. Because now we can refer to an ophthalmologist if needed, but what do we do to rehabilitate?* (G4)

Most of the participants emphasised that even though their competence for vision assessment was not on an expert level, doing a basic assessment was much better than doing no assessment.

*Participant 1: ‘You can’t do anything wrong by doing the assessment; you will identify large vision problems’.*

*Participant 2: ‘That’s right, I agree. It is much better that someone actually does a vision assessment’. (G3)*

#### More appropriate in a rehabilitation setting

Although the participants from home care said that they intended to use the KROSS tool, they had a few new stroke patient in their services. They agreed that assessing vision after stroke was important but reflected that the assessment was more appropriate to be performed in other municipal services, such as the rehabilitation unit and home rehabilitation where they have a more explicit focus on functional assessment of their patients.

*That’s when I’m thinking about the rehabilitation unit, right? I am thinking that this [KROSS assessment] is a very good thing when patients are in inpatient care. You know, there are many assessments and tasks that you should do, but they can’t be all done in an hour. You have to do it at different times and when you find it appropriate. We [home care] are just in and out, but in the rehabilitation unit, they have the patients all 24 hours (G1).*

### Feasibility

Four categories represent the participants’ experience of the feasibility of implementing the KROSS tool in their setting. Being new to vision assessment, the participants discovered that they needed time to get familiar with performing, interpreting and documenting their assessment. They thought the available instructions were good, especially when combined with supervision, which was helpful when starting to use the KROSS tool.

#### Practise makes perfect

Because many of the different tests in the KROSS tool were new to the participants, they needed time to familiarise themselves with the tests to perform them properly with different patients. In periods where they could do the assessment regularly, they experienced the tests as easier to perform and felt more skilled and confident in performing the assessments.

*Initially, I made many mistakes. I had to do some tests several times. Forgot to ask them to cover one of the eyes and such. (G4)*

*It’s like, if you have done it one week, and the next, several times in a row, you feel more confident. Then again, if it’s a month since the last time, you get unsure again.* (G2)

#### Helpful instructions and supervision

Most of the participants thought the instruction manual for the assessment was easy to use and understand. All used the manual during the assessment, and some read the manual before to prepare themselves and thought that doing so improved their performance. The opportunity to ask questions or get supervision in their practice during the implementation was helpful, especially in the initial implementation.

*It’s reassuring to get help if there is a challenging assessment. That we can send an email to the project group so they can do an additional assessment (together with us). That is a reassuring for us and the patients.* (G3)

#### Integration of the KROSS tool into the medical records ease documentation

In the municipality, they had integrated the KROSS tool into the medical record. The KROSS results were stored in the patients’ medical records. This made it easy for other HCP in the municipality to find the test and read the results of the vision assessment.

*The best way is to plot the results right into the electronic form. Just tick it off. (G3)*

The specialist services had not integrated the KROSS tool into their medical records and struggled to describe the result from the assessment in words. This was because of a lack of knowledge about the terms and expressions used to describe visual function. Some suggested producing a standard text that they could adapt to each patient.

*I think that it can be difficult to get it in the medical record in a sensible way. Because it ends up with long dissertations because I don’t know the right name on the different test, and it’s hard to write it in an easy way.* (G4)

#### Limited time available

For those who did not perform the KROSS tool as intended, a lack of time was one important explanation. This was particularly true for home care. Although some of the services assessed most patients, sometimes the personnel with KROSS assessment training were not available or had to prioritise other tasks, which meant that some patients were not assessed.

*I guess it is a thing that I, at least in ****my ****workday, can find the time (to test vision with the KROSS tool). I just have to rearrange my schedule.* (G2)

### Fidelity

Although there was no formal evaluation of the participants’ assessments to measure fidelity and accuracy in the present study, it was an aim that all stroke patients should be assessed with the KROSS tool. Most of the participants expressed that they used the KROSS tool and followed the instructions as intended; however, some said they did not test all the patients.

#### Followed the KROSS protocol but did not test all patients

Even though some items in the KROSS tool, for example, assessing the visual field, were experienced as complex, especially in the beginning, the participants said they always completed the whole test with all the items included. The aim was to test all patients who were diagnosed with a stroke, but some participants only tested patients when they suspected a visual problem. This was discussed between the participants as problematic because there may not be any obvious signs of VIs.

*Participant 1: But testing all? We do not do that. But I think it has been really good to use when we suspect a visual problem. Earlier, we did not have a tool to test vision with, and we just tried to separate VIs from other impairments.*

*Participant 2: But will you identify all patients with VIs if you don’t test all, or? (G4)*

### Penetration

Three categories from the data were related to penetration. The KROSS vision assessments were also requested by HCP who not had been a part of the KROSS workshops; here, vision became a part of the observations of their patients, and it improved the planned follow-up of VIs after stroke in the health care services.

#### Vision assessment included in service allocation office case handling

All the participants had become more aware of the importance of vision assessment after a stroke. Usually, handling cases in this municipality mostly specified the right service level rather than details about the content of the services. Participating in the KROSS project had resulted in the case handlers who were working in the service allocation office now beginning to ask the service providers to perform the KROSS assessment when the municipal received new stroke patients from the hospital. Thus, a vision assessment had become an area in which they specifically instructed service providers to consider.

*In some cases, the service allocation office has asked us to do a KROSS test while the patient is in rehabilitation. They put it in the order. That is very good. (G2)*

*Now, asking the services specific for vision assessments is something more than we usually do as case handlers. …. Mostly, we just decide on the level of the service and its main content.* (G3)

#### Visual function assessment integrated into the clinical awareness

All the participants said that they now paid more attention to vision and visual impairments in general. They were considering vision when they observed their patients in different situations, such as ADL and mobilisation. Vision became more integrated in their clinical gaze when caring for their patients. Some found it helpful to use the KROSS tool to assess vision in patients without stroke as well.

*I have also done the assessment (KROSS) on a patient without stroke who had terrible vision. I became curious and wondered, ‘How bad do you see? Or do you struggle with other impairments?’ It turned out that he saw just terribly, poorly. Then, we were able to do something about it. (G2)*

#### More structured interdisciplinary collaboration with vision experts

As a result of participating in the KROSS KT project, awareness and attention to VIs were increased. The specialist rehabilitation hospital had also started to collaborate with an optometrist who could assess patients at the hospital. The participants considered this a significant improvement compared with previous vision care but would prefer a more permanent solution with a vision expert integrated in their service. The participants also expressed that they had now increased their knowledge about different vision rehabilitation services and referred more patients to vision rehabilitation.

*We have had optometrists here to assess patients in our hospital. We never had that before this project, and I think we have referred more patients to vision rehabilitation than we did before.* (G3)

### Sustainability

In different ways, the KROSS assessment routine was integrated into already existing routines in the services. The participants’ became more aware of the need for more competence regarding vision impairments, and to enable further improvement in future vision care, they wanted further formalised vision education.

#### Integration into existing routines

The rehabilitation unit had included the KROSS vision assessment as a part of its existing whiteboard routines. On the whiteboard, all important activities or assessments for each patient were listed [30]. The whiteboard list was used as a checklist and topic agenda for their multidisciplinary meetings; now, the KROSS assessment was also included on the whiteboard.

*We now have an item on our whiteboard where it says: KROSS test. This is part of the total assessment package. We mark the task with a red button, so it is how we control that we secure follow-up.* (G2)

Six months after the KROSS KT project started, the municipal rehabilitation unit moved to a new location. With a new office and new whiteboard, the participants said that the KROSS assessment was still included and integrated in their routine service.

Another way that the municipality had promoted sustainability was that the KROSS tool was integrated into their medical record system. Still, some of the participants expressed that the most practical aspect for them was to have a paper version to bring to the bedside or the patients’ home and later transfer the results to the medical record. The specialist rehabilitation hospital had included the KROSS vision assessment as part of their formal routine for all stroke survivors as part of their baseline assessments.

#### Desire for formal vision competence

After having some experience with the KROSS tool, the participants acknowledged that they needed more knowledge and a better understanding of visual function when doing the assessment.

*Compared with other things we are assessing, we barely have competence in assessing it [vision]. (G3)*

Several wanted more formal vision competence, for example, a continuing education course or even a master’s degree. They wished they had learned more in their professional education and wanted vision to have a higher priority when new HCP were educated.

*Before the KROSS workshop, I did not know anything about VIs after a stroke. I knew it existed, but in my education, we did not learn anything about it. (G4)*

Some wanted to be able to do a more comprehensive assessment but also to have the competence to start vision rehabilitation.

*After participating in this project, I am thinking about possible rehabilitation options for VIs. Is there a course, education or anything that we can take or something?* (G3)

## Discussion

The current study produces important new knowledge about the implementation of structured vision assessment into health care services by HCP without vision expertise. The results show that it is possible to integrate a structured vision assessment with the KROSS tool but that the level of integration depends on how well the implementation is tailored to the local context and accepted by all users and stakeholders [20, 24]. All the participants expressed they found it acceptable to include the KROSS vision assessment in their practice; they were motivated by the experience that knowledge about the patient’s visual function was helpful for training ADL and other rehabilitation activities. The KROSS tool was adopted in most of the services, except for those working in home care who had not been able to do so. This also influenced the participants’ experiences of how appropriate it was to use the KROSS tool in their services. Integration in the services’ existing routines and systems [31], together with a motivation for gaining additional knowledge and better routines for vision after stroke, were the facilitators for a sustainable change of practise [32].

After participating in this implementation project, the participants expressed a high level of acceptance of the KROSS tool and the new structured vision routines. They highlighted that the content of the KROSS workshop was directly useful for their practice and were motivated by knowledge about the potential consequences of VIs after stroke. Experiencing improvements for service users is important for acceptability [33], and the participants stated that assessing vision was now seen as important to include in their practise. The current study indicates that being provided with the KROSS tool, in combination with experience of the benefits of identifying present VIs, influenced the participants’ perceptions of acceptability.

Even 16–18 months after the KROSS workshop, the participants still valued the importance of knowing about their patients’ visual function, which is considered an important facilitator for sustainable change [26]. Their acceptability was initially related to the expected positive impact for their patients, which was later confirmed for those who adopted the KROSS tool in practise because knowledge about the patient’s visual function helped them perform better as HCP. This experience motivated the participants to continue to use the KROSS tool. Several studies have shown that an experienced beneficial change of practice increases the probability of adopting a new routine [20, 34, 35]. Motivation is important for changing practise [36], and the participants in the present study maintained a high level of motivation throughout the project. Proctor et al. described that acceptability can change over time. Something experienced as acceptable when being presented for the first time can be less acceptable after using it in practise [26]. In the current study, however, the participants expressed a high level of acceptability throughout the project.

### Different levels of integration of the KROSS tool

Although most of the participants said they had adopted using a vision assessment with the KROSS tool as part of their routines, there were variations between the services. The participants in the rehabilitation services and stroke unit stated that they could start using the KROSS tool immediately after the KROSS workshop and had integrated it into their daily routines. Home care initially intended to use the KROSS tool in practice, but with a lack of patients, they never managed to adopt it. Intention for change, as all reported, is an important precondition for actual change. However, as other studies have found, many do not manage to change their behaviour, even if the initial intention is strong [26, 37]. The current study has found a lack of adoption in home care, even though they thought the implementation was both acceptable and feasible. The reason given by the participants was that they had not seen any stroke patients. When they were not able to use their knowledge and practice their skills right away, this might have reduced their attention towards adopting the implementation. The lack of stroke patients was unexpected because it is reported that 20% of stroke survivors receive help from home care 3 months poststroke [23]. It is possible that several of the stroke survivors had already received rehabilitation before they moved home, either in an institution, in an outpatient rehabilitation or by the home rehabilitation team [1, 6, 23]. This was emphasised by home care HCP as an explanation for why they thought it was more appropriate that vision should be assessed earlier in the stroke care pathway.

Being generalists and not stroke specialists was identified as a barrier to using the KROSS tool before implementation [20]. This means that if one focuses on a specific condition or diagnosis in a service, it will be easier to see the need for improvements and adopt new knowledge in practise [20]. The HCP from the rehabilitation hospital and municipal rehabilitation services consider themselves stroke care and rehabilitation specialists. In a Norwegian context, home care are generalists, traditionally concerned about helping patients with their daily living [20, 38], without a formal responsibility for rehabilitation, leaving this up to other services [39, 40]. This might have influenced their experiences of their capability to perform the assessment properly, which is important for implementation [36]. The HCP accepted that it is important to assess vision in stroke care but felt home care services were not the most appropriate service. This suggests that future implementation needs to consider all stroke services as a continuum of care and find a way to ensure that all stroke survivors have their vision assessed either in the stroke unit or in the rehabilitation services before receiving home care.

Although we had already assessed the barriers and facilitators before the implementation [20], we identified some new barriers in the present study. One barrier was that it was a challenge to stay in touch with home care services after the KROSS workshop. Interestingly, they did not use email on a daily basis, and a second barrier was the large staff turnover. This made it difficult to support the home care participants by sending reminders and information, visiting them in practise for supervision and providing feedback, which were important implementation strategies. We had planned for follow-up and supervision for all services but did not manage to include home care as intended. Home care HCP reported a high level of workload and small opportunities to plan and prioritise their workday. Experiences of limited resources and structural organisational barriers are important contextual determinants for implementation [41]. In the current project, we did not have any additional resources to add to the services, which might have affected adoption.

### Keeping it simple while still performing an adequate vision assessment

In the current study, we found that after putting it in practise, the KROSS vision assessment tool was experienced as feasible. With some experience, time use was reduced, and the participants felt more confident with the assessment procedure and less dependent on the user manual; they were also offered supervision in their own practise. This was emphasised as important, especially in the initial phase of the implementation. Indeed, sufficient training and competence for performing an intervention is important for the experience of feasibility [26].

After conducting several assessments in practise, some of the participants wished they had a more comprehensive competence in vision assessment and rehabilitation. Although some expressed concern about a lack of specialised vision competence in their services, all groups agreed that assessing vision is an important first step to improve vision care. This is important because the first step in helping someone with a vision problem is identifying its presence [18, 19, 42, 43].

### Vision assessment should be included in the care pathway for all stroke survivors

In terms of fidelity, the current study showed that when testing the patients, the whole KROSS tool was used, not just some parts of the assessment. Although most of the participants had ambitions to test all stroke survivors, others said that they only tested the patients if they suspected VIs. The intention with the routine was to test all patients because of the difficulties in identifying VIs without a formal vision assessment. Assessing vision only on those suspected of VIs may leave some patients with a possible vision problem going undetected [14, 42]. The nature of vision problems requires a formal vision assessment of visual acuity, eye movements, visual attention and visual field [16, 18, 42]. It is necessary to communicate this more clearly to ensure that all patients receive a vision assessment.

In the UK, a stroke–vision pathway has been developed based on a consensus study by Rowe et al. [43]. In this pathway, the authors suggest that a well-defined pathway for vision assessment and rehabilitation, together with support services, should be integrated into stroke services. Depending on when and where the patients present their vision symptoms to HCP, there should be a procedure to provide vision care. In stroke services without immediate access to vision specialists, Rowe et al. recommend the use of vision assessment tools to identify a possible vision impairment to secure a proper referral to vision care [43]. As emphasised in the current study, it is crucial that HCP working with stroke survivors are aware of VIs as a possible symptom or sequela of stroke; in this implementation study, we see that the participants are more aware of VIs and that most of them use the KROSS assessment tool. To promote a multidisciplinary approach for stroke survivors with VIs, a vision specialist should be integrated in the multidisciplinary stroke team [44]. This could add to a better understanding of the stroke survivor’s functional vision and how an impairment can affect other functions. HCP without vision competence would learn from HCP with vision competence and vice versa [44].

### Integrating the assessment tool into existing routines and systems for sustainable implementation

In municipal health care services, case handlers from the service allocation offices joined the implementation because we believed it was important that they knew about the project. The case handlers requested a vision assessment in their description of the patient’s service decision. This was the participants’ independent initiative resulting from increased attention to VIs after stroke and is a reminder of the importance of involving a larger part of the organisation than just the HCP working closely with the patients. Other studies have described that involving several parts of the organisation and leaders are possible determinants for the sustainability of an intervention [45].

In the current study, we found that integrating the new procedures into existing routines was effective, such as including the vision assessment with KROSS on the whiteboard and whiteboard meetings. Preparing an infrastructure for new interventions, such as a new tool, was important for sustainable use. Things that are separate from the already established routines and come as an additional new task may need additional attention from HCP, making it easy to forget [46]. In addition, we found that storing the assessment form with the results from the assessment was the preferred way to document their findings. Documenting the results of the vision assessment in their own words was challenging because of the lack of a precise language to describe visual function.

The strategies to increase knowledge and skills about VIs during the implementation increased the participants’ capability to perform the vision assessment. Performing the vision assessment and experiencing its importance for their patients influenced their motivation for continuing to improve their capability to provide proper vision care. We believe that the improved capability positively affected the motivation of the participants, and those who used the KROSS tool were further motivated by its significance for their patients. This is supported by other studies showing that motivation and experiencing that the implementation has positive consequences for their patients can facilitate sustainable change [36, 45].

### Small investments for better vision after stroke care

Cost was not formally assessed in the current study. There was no need for additional equipment, and the direct costs were related to the need to replace HCP so they could attend the KROSS workshop. One thing related to the use of resources was the time it takes to perform the KROSS assessment in practise. The participants had reported a tight time schedule before the implementation, so they were asked about their experiences of adding the KROSS assessment in their practise. The participants who used the KROSS tool said that they had to prioritise the use of the tool within their own workday at the expense of other important work. However, they found that the benefit of the vision assessment outweighed the cost of time. Changes experienced by the HCP as beneficial for patients are more likely to be successfully implemented [34, 35]. Further specifying of the time it takes for an experienced user to perform the KROSS assessment should be done to prepare other health care organisations for implementing the KROSS tool.

### Strengths and limitations

There are many ways to evaluate implementation projects. In this project, we found that a qualitative evaluation of the participants’ experiences of using the KROSS tool in their own practise was more appropriate than a quantitative study of knowledge use or feasibility [47]. The results are representations of the participants’ experiences, which are expressed in focus group interviews; here, for instance, adoption may be overestimated by the participants. However, we had contact with the HCP during the implementation and supervised them in practical testing. In addition, many patients were referred to the university’s clinic for further assessment. The home care group consisted of only two participants, and they had not used the KROSS tool since trying it out in the KROSS workshop. This means that they had little experience with the implementation to share. Therefore, the material represents home care only in some of the implementation outcomes, and this should be considered when interpreting the results.

The KROSS KT project began as an implementation project for municipal health care services. During the start-up process and cooperation with the municipal and user organisations, we were asked for participation from other health care services. We chose to involve the participants from a specialist rehabilitation hospital and acute stroke unit. Although they were offered follow-up, they did not receive the same amount of attention after the KROSS workshop but reported to have integrated the KROSS tool into their routine patient care. Even if the services outside the municipality received less attention in the planning and the follow-up phase, they were eager to participate when they heard about the project. Their participation in the KROSS workshop and this evaluation have contributed to the KROSS KT project with valuable insights; that is, showing that the project has expanded and involved other services than first planned is an example of penetration.

In the implementation, we developed multifaceted strategies to engage the participants and promote knowledge use [25]. This makes it difficult to single out the strategies that worked and those that did. We believe that the combination of strategies was important to recruit, motivate and engage the participants for behaviour change. If the KROSS tool should be used in a different setting or context, the variety of implementation strategies should be addressed to barriers and facilitators specific to the context, as recommended in the KTA model [24]. Engaging the health care organisation, including leaders, case handlers and bedside HCPs, in the implementation was important for the results in the current study.

## Conclusion

The participants found the KROSS vision assessment acceptable for use in their practise and were motivated by using it because they experienced it as beneficial for their patients. Although most of the participants had included KROSS in their services, home care had not been able to do so. They considered that rehabilitation services would be most appropriate for structured vision assessment because of the limited number of stroke patients they see and the organisation of their workday. Assessing vision was new to most of the participants, and it appeared important to improve theoretical knowledge and practical skills in vision assessment. The enhanced vision competence led to increased collaboration with vision experts and referrals to vision rehabilitation and, in some cases, a motivation for obtaining more and formalised competence in vision care and rehabilitation. To facilitate better vision care after stroke, vision assessment and follow-up should be included in the care pathway description and be integrated in services that provide stroke care.

## Supplementary Information

**Additional file 1.** Interview guide.

## Data Availability

The transcripts and notes used and analysed during the current study are not publicly available due to protection of the anonymity of the participants, and the content may threaten confidentiality. An anonymised version of the data can be made available from the corresponding author on reasonable request.

## Abbreviations

COREQ: Consolidated Criteria for Reporting Qualitative Research
HCP: Health care personnel
KT: Knowledge translation
KROSS: Competence, Rehabilitation of Sight after Stroke
KROSS KT: KROSS knowledge translation
KTA: Knowledge to action
VIs: Visual impairments
VISA: Vision impairment screening assessment
