# Supplementary material for: ‘If we don’t assess the patient’s vision, we risk starting at the wrong end’: a qualitative evaluation of a stroke service knowledge translation project
Source: BMC Health Serv Res. 2022 Mar 16;22:351. doi: 10.1186/s12913-022-07732-w (PMC8925164; doi:10.1186/s12913-022-07732-w)
Supplement: Supplementary file 1 — Additional file 1. Interview guide. [file 12913_2022_7732_MOESM1_ESM.docx]

# Interview guide

Short presentation of the study, and what the information collected in this focus group would be used for. Inform of the ethical considerations, that participation is voluntary and that it is possible to withdraw anytime. Information and getting consent to audio record. Ask if any questions before signing of the informed consent form.

*Start audio recording*

| Theme | Question |
| --- | --- |
| Role and responsibility in stroke care | Which health care service do you work in? Workplace, profession and experience. |
| Introduction | Experiences with being involved in the KROSS KT project after the implementation.   - How are your experiences of using the KROSS procedure? - How does the patient respond to the vision assessment? - What are the responsibility of your service related to vision care and rehabilitation after stroke - If this project should be repeated elsewhere, do you think your service should be included? - How are your experiences of your leader’s involvement in the KROSS KT project |
| Experiences with using the KROSS tool | Using the KROSS tool   - Have you used the KROSS tool in practise? - If not, elaborate on why? - If yes, elaborate on how you use it? - Who does the vision assessment? (other than you? Profession/How many?) - Experiences of documenting vision assessment - Is it easy to find Previous KROSS assessments in the medical journal? - How do you document the findings? How do you store the assessment tool?   Time   - Approximately how much time do you use to perform a KROSS assessment? - Do you think the time it takes is acceptable?   Complexity   - Do you find perform the assessment difficult/easy? - How do you find the user manual?   Reflections on adoption/non adoption   - Insecurity of the assessment procedure? - Difficult to get in a routine? - Elaborate reasons why/why not |
| Experiences with the KROSS KT project | For those of you who participated in the KROSS workshop, how did you experience it?   - Was there sufficient follow up and supervision after the workshop? - Do you think competence about vision is important for your profession and services? - What is important for you to continue/start to assess vision? - All things considered, has participation in the KROSS KT project been worth the effort? - For those not participating in the workshop, how are your experiences with the KROSS KT and how did you get involved in being an active user of the KROSS tool?? |
| Practical organisation | - How do you organise the KROSS assessment, how is it organised in your service? - Do you use a stroke care pathway or written routine for stroke patients? |
| External collaboration | - How do you inform other services about the vision assessment? - Have the KROSS KT project had any influence on how you plan further rehabilitation for your patients? |
| Finally | Are there anything you want to add to this discussion that we have not addressed in this focus group? |
